# High-Strength Ultrafine-Grained Al-Mg-Si Alloys Exposed to Mechanical Alloying and Press-Forming: A Comparison with Cast Alloys

**DOI:** 10.3390/ma18010099

**Published:** 2024-12-29

**Authors:** Wenjie Zhong, Lin Song, Huaguo Tang, Xu Wu, Zhuhui Qiao, Xunyong Liu

**Affiliations:** 1 Shandong Laboratory of Advanced Materials and Green Manufacturing at Yantai, Yantai 264006, China; zwj15684139786@126.com (W.Z.); laotang@licp.cas.cn (H.T.); amgm_wuxu@163.com (X.W.); 2 School of Chemistry and Materials Science, Ludong University, Yantai 264025, China; 3 Yantai Zhongke Research Institute of Advanced Materials and Green Chemical Engineering, Yantai 264006, China; 4 State Key Laboratory of Solid Lubrication, Lanzhou Institute of Chemical Physics, Chinese Academy of Sciences, Lanzhou 730000, China; 5 Yantai Huihua Metal Sci. & Tech. Co., Ltd., Yantai 264006, China

**Keywords:** Al-Mg-Si alloys, high strength, mechanical alloying, press-forming, grain refinement, stacking faults

## Abstract

A high-strength Al-Mg-Si alloy was prepared using mechanical alloying (MA) combined with press-forming (PF) technology, achieving a strength of up to 715 MPa and a hardness of 173 HB. The microstructures were comparatively analyzed with conventional cast Al-Mg-Si alloys using XRD, TKD, and TEM. The XRD results showed that the full width at half maximum (FWHM) of the alloy prepared by MA+PF was significantly broadened and accompanied by a shift in the diffraction peak. TKD revealed that the grain size of the MA+PF processed alloy was significantly reduced to approximately 260 nm, indicating substantial refinement compared to the cast alloy. Additionally, using TEM, it was found that the newly developed MA+PF alloy exhibited a distinct morphology of Mg_2_Si precipitation phases and a high density of stacking faults (SFs), characteristics that differed from those in the cast alloy. The significant enhancement in strength can be attributed to the synergistic strengthening effects of grain refinement, second-phase precipitation, and stacking fault strengthening, as synthesized and analyzed.

## 1. Introduction

The Al-Mg-Si alloys (6xxx series aluminum alloys) are ideal for manufacturing complex structural components due to their low density, high specific strength, excellent corrosion resistance, and remarkable malleability. They are extensively utilized in various industries, including aerospace, rail transportation, construction materials, and automotive parts [1,2,3].

With the widespread implementation of energy conservation and environmental protection regulations in recent years, the trend towards lightweight components has led to a significant increase in the demand for lightweight materials. However, the existing Al-Mg-Si alloys are struggling to meet the increasingly stringent strength requirements. Therefore, there is an urgent need to develop novel Al-Mg-Si alloys with higher strength and toughness. Currently, a prevalent strategy to improve the mechanical properties of Al-Mg-Si alloys involves incorporating additional alloying components, including transition metals, rare earth elements, and further modifiers into the composition [4,5,6]. Bosio et al. [7] demonstrated that adding 4 wt. % of Cu into Al-Mg-Si alloys increased their yield strength by 35.6%, achieving approximately 430 MPa. This enhancement was attributed to the improved solid solution and precipitation strengthening mechanisms induced by Cu atoms. Xu et al. [8] discovered that the F357 alloy developed a fine Al_3_Sc strengthening phase with the addition of 0.8 wt. % Sc, resulting in a tensile strength of 356 MPa. Rahimian et al. [9] investigated the effect of Zr on the structure and properties of Al-Mg-Si-Cu alloys, noting the formation of 80–200 nm Al-Si-Zr-Ti phases and strengthened *θ*′ and *Q*′ precipitates. However, when enhancing strength through compositional modifications, there is a risk of instability, making the material susceptible to solid solution diffusion, which can lead to material failure or only marginal strength improvements.

Grain refinement is regarded as an effective method to enhance the strength of aluminum alloys [9]. The finer the grains in an alloy, the more numerous the grain boundaries become, effectively impeding dislocation motion and slip, which significantly improves strength [10,11]. Refining grains to the nano-scale can greatly enhance the overall properties of aluminum alloys [12]. Furthermore, stacking faults (SFs) are prevalent planar crystalline defects in metals and alloys. On the one hand, SFs can impede the movement of dislocations; on the other hand, they can serve as storage sites for dislocations, promoting their accumulation. These two effects can considerably improve the strength and ductility of alloys [13,14]. However, aluminum has a high stacking fault energy (SFE) of 166 mJ/m^2^ [15], which is only weakly influenced by alloying elements, making the formation of SFs challenging in aluminum alloys [15]. It has been reported that a fine grain size can lower the SFE and induce the formation of SFs in alloys [16,17]. The accumulation of plastic strain resulting from extensive plastic deformation can also promote the formation of SFs in aluminum alloys. Therefore, achieving grain refinement is not only essential for the formation of SFs in aluminum alloys but also crucial for realizing the synergistic effects of fine-grain strengthening and SF strengthening, significantly improving the overall strength of aluminum alloys.

The mechanical properties of cast Al-Mg-Si alloys are significantly influenced by macroscopic defects, such as shrinkage and porosity, along with compositional segregation issues stemming from uneven element distribution [18]. Heat treatment processes can improve the strength of casting Al-Mg-Si alloys, but they still fail to meet the current demand for high-strength alloys in various fields [19,20]. Modifying alloy elements to enhance the strength of casting Al-Mg-Si alloys is also a common approach, yet it presents drawbacks such as unstable effects and unclear improvements [21,22]. Therefore, there is an urgent need for a new process capable of achieving grain refinement combined with SF reinforcement, thereby enhancing the overall strength of aluminum alloys.

Distinguished from the complexities of traditional casting processes, this method circumvents the poor surface quality and mechanical properties often associated with conventional castings. Mechanical alloying (MA), as a solid-state process technology, facilitates the formation of nanocrystalline compounds and alloys through non-equilibrium processing methods [23,24]. Research has shown that during high-energy ball milling, the alloying elements are continuously cold-welded and crushed under the action of the grinding balls, resulting in a high degree of grain refinement. Simultaneously, the collision of the grinding balls provides impact energy for the alloying elements, offering the possibility of inducing the generation of SFs [25]. The press-forming (PF) process is a rapid sintering and shaping method conducted under pressure, capable of achieving dense powder sintering swiftly. This method facilitates the preparation of high-strength aluminum alloys with nano- or submicron-scale structures [12]. Tang et al. [12] prepared high-strength Al-Cu-Mg alloys with a tensile strength of up to 780 MPa and microhardness reaching 180 HV using the MA+PF method. Moreover, this process has the potential to be straightforward and suitable for large-scale industrial production. Therefore, it is considered effective for achieving grain refinement and SFs, using MA+PF to attain a substantial increase in the overall strength of Al-Mg-Si alloys.

In present work, we produced ultrafine crystalline aluminum alloy grains using MA+PF, while inducing SFs in the aluminum matrix. The goal of this preparation is to achieve high-strength aluminum alloys through a synergistic strengthening mechanism that combines fine-grain strengthening with stacking fault strengthening. Furthermore, the microstructure and mechanical properties of Al-Mg-Si alloys prepared using the MA+PF technique (denoted as MPA) were compared with those of Al-Mg-Si alloys created by casting (denoted as CA).

## 2. Experimental

### 2.1. Material Fabrication

**Figure 1:**
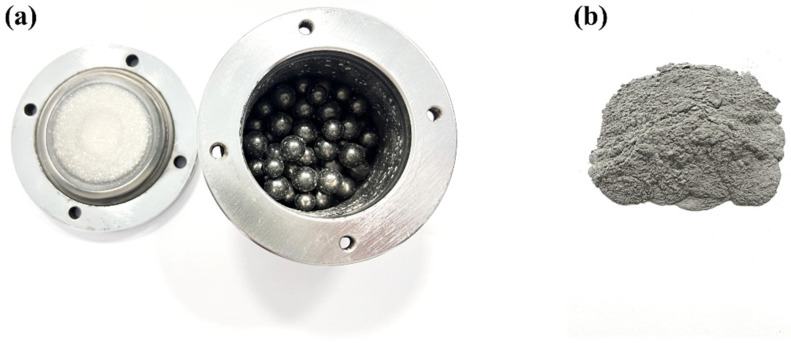
(**a**) Ball milling jar; (**b**) mixed powder after ball milling.

Powders of 94.4 wt. % Al (380 mesh, 99.7% purity), 4.0 wt. % Mg (180 mesh, 99.7% purity), and 1.6 wt. % Si (100 mesh, 99.7% purity) were used as starting materials for the mixed powder. The mixed powders were placed in a steel ball milling jar containing 168 g of steel balls (the mass ratio of balls to powders is 12:1) in Figure 1a. To prevent the oxidation and cold-welding of powders during ball milling, 112 μL of acetone and 140 μL of ethanol were added to the ball milling jar as process control agents (PCAs). The PCAs can form a protective film on the Al and Mg powder surface, effectively inhibiting excessive oxidation and slowing down the high-energy impact from grinding balls to the powder [26,27,28,29]. Therefore, the MA process can be safely carried out under conventional atmospheric conditions without argon protection. Subsequently, ball milling experiments were conducted to prepare highly reactive Al-Mg-Si alloy powders using a GN-2 high-energy ball mill (Shenyang Oute Vacuum Technology Co., Shenyang, China) set to a rotational speed of 600 rpm for 6 h at room temperature, the mixed powder after ball milling is shown in Figure 1b.

**Figure 2:**
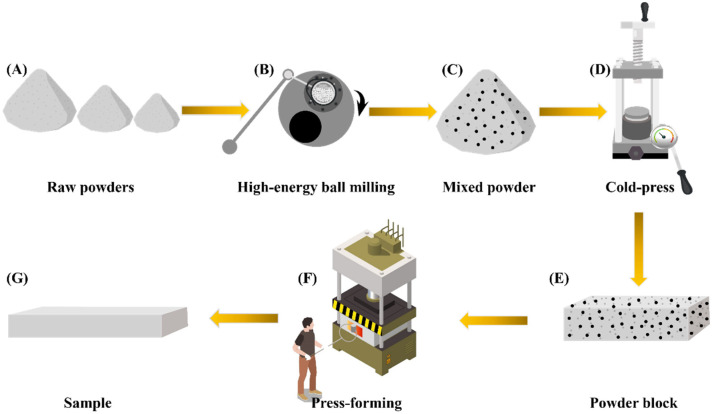
Schematic diagram of the complete process. (**A**) Raw powders, (**B**) high-energy ball milling, (**C**) mixed powder, (**D**) cold press, (**E**) powder block, (**F**) press-forming, (**G**) sample.

The Al-Mg-Si alloy mixed powder, after high-energy ball milling, was pressed into a blank measuring 60 mm × 8 mm × 12 mm under a pressure of 400 MPa and subsequently wrapped with graphite paper. The graphite paper-wrapped blank was placed in a pre-heated hot-worked die steel mold at 580 °C for 3 h and held for 3 min, before being pressed at a pressure of 400 MPa within 30 s. The Al-Mg-Si alloy product, sized 75 mm × 6 mm × 12 mm, was removed and cooled to room temperature. The schematic diagram illustrating the entire process is depicted in Figure 2.

The 94.4 wt. % Al-4.0 wt. % Mg-1.6 wt. % Si alloy was transferred to an electric resistance furnace and held at 750 °C for 20 min. Subsequently, the melt was poured into a preheated mold (approximately 200 °C) to obtain an Al-Mg-Si alloy casting product.

### 2.2. Microstructure Characterization and Mechanical Property Test

X-ray diffraction (XRD) utilizing Cu Kα radiation at 40 kV and 100 mA (Bruker D8 ADVANCE) was employed to analyze the physical phases of Al-Mg-Si alloy powders, CA, and MPA, following high-energy ball milling. Field emission scanning electron microscopy (FESEM, TESCAN CLARA GMH) was used to characterize the particle morphology and microstructure, as well as the fracture surfaces, of the tensile specimens. The distribution patterns of the Al matrix and generated phases (e.g., Mg_2_Si) were characterized using high-resolution transmission electron microscopy (HRTEM, FEI Talos F200X), and the elemental distributions of the precipitated phases were statistically detected by EDS. The crystallographic orientation and grain size of the Al phase were analyzed using transmission electron microscopy (TEM, FEI Talos F200X) equipped with a transmission Kikuchi diffraction detector head (OPTIMUS TKD).

**Figure 3:**
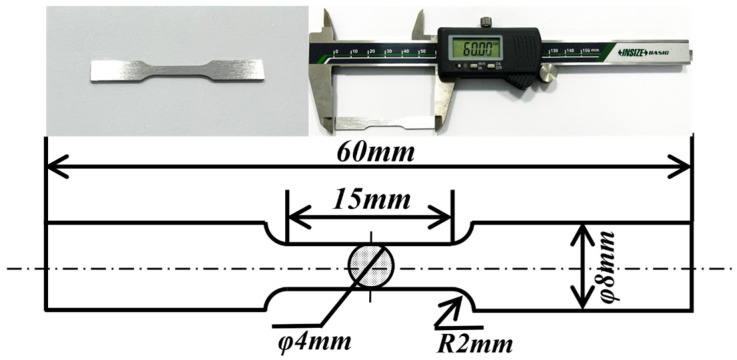
Tensile specimen and schematic diagram of tensile specimen.

To evaluate the ultimate tensile strength (UTS) of CA and MPA, all specimens were machined into dumbbell-shaped tensile specimens with a cross-sectional width of 3 mm, a gauge length of 15 mm, an overall length of 60 mm, and a thickness of 2 mm, as depicted in Figure 3. Room temperature tests were conducted using an Instron 68FM-100 tensile testing machine at a constant strain rate of 1 mm/min. Stress–strain curves were recorded separately for the CA and the MPA specimens. Additionally, microhardness test specimens were machined to dimensions of 25 mm × 5 mm and evaluated using a Brinell hardness tester (HBS-30000MZ) under a 50 N load for 30 s at room temperature. Each specimen underwent at least three random individual measurements to determine an average value.

## 3. Results and Discussions

### 3.1. Powder Characterization

**Figure 4:**
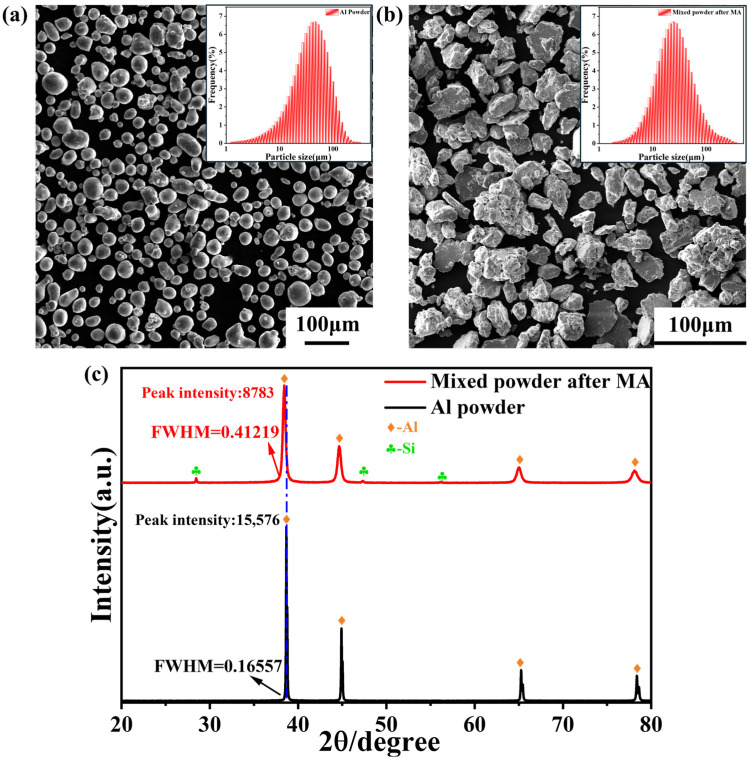
SEM images and particle size distribution of various powders: (**a**) Al powder, (**b**) mixed powder after MA, and (**c**) comparison of typical XRD patterns of Al powder and mixed powder after MA.

From Figure 4a, it is evident that the powder morphology of raw Al powder without MA exhibits a spherical or olive shape. The particle size, illustrated in the inset of Figure 4a, indicates a uniformly distributed overall particle size, with an average of 41.2 μm. Following intense MA, the morphology of the mixed powders is depicted in Figure 4b. The overall appearance of these mixed powders is irregular, primarily consisting of flattened flakes or lumps. Furthermore, their particle size distribution is more uniform, with an average size of 26.2 μm, as depicted in the inset of Figure 4b, compared to the raw Al powders that were not subjected to MA.

It can be clearly observed in Figure 4c that the intensity of the main diffraction peak of the mixed powder after MA treatment is significantly reduced to 8783 a.u., indicating a notable decreasing trend compared to the intensity of the main diffraction peak of the pristine Al powder. The reasons for this phenomenon involve two key factors. First, during the MA process, the Mg and Si atoms penetrated the lattice structure of the Al matrix, leading to the formation of a solid solution. The presence of these foreign atoms in the solid solution disrupts the original lattice arrangement of the Al matrix, resulting in a decrease in the crystallinity of the Al matrix, which is reflected in the X-ray diffraction pattern as a decrease in the intensity of the diffraction peaks. Second, the MA process is often accompanied by the introduction of numerous crystal defects, such as dislocations. These defective structures scatter the incident X-rays, further weakening the intensity of the diffraction peaks [30]. Additionally, we also noticed that the half-peak width of the diffraction peaks of the MA-treated powders exhibited a significant broadening phenomenon. This change is primarily caused by grain refinement and distortion of the lattice due to MA action during ball milling [31]. Further in-depth observations reveal that the positions of the diffraction peaks shift towards the low-angle direction, strongly indicating that the Mg atoms with large atomic radii have successfully diffused into the Al matrix and formed the α-Al phase. It is also noteworthy that in the XRD spectra of the MA-treated mixed powders, only the diffraction peaks of Al and Si were observed, while no peaks of any other monolithic or non-homogeneous phases were detected.

In summary, the characteristics of the MA-treated mixed powders, including the decrease in diffraction peak intensity, the increase in half-peak width, and the shift in diffraction peak position, collectively indicate the significant impact of the MA treatment on the mixed powders. Specifically, this process has led to a considerable degree of grain refinement of the mixed powders and the diffusion and solid solution of all elements except Si into the Al matrix. Meanwhile, at the end of the MA treatment, the residual Si elements remain in the amorphous state and do not introduce a completely solid solution into the matrix.

### 3.2. Microstructure Analysis

**Figure 5:**
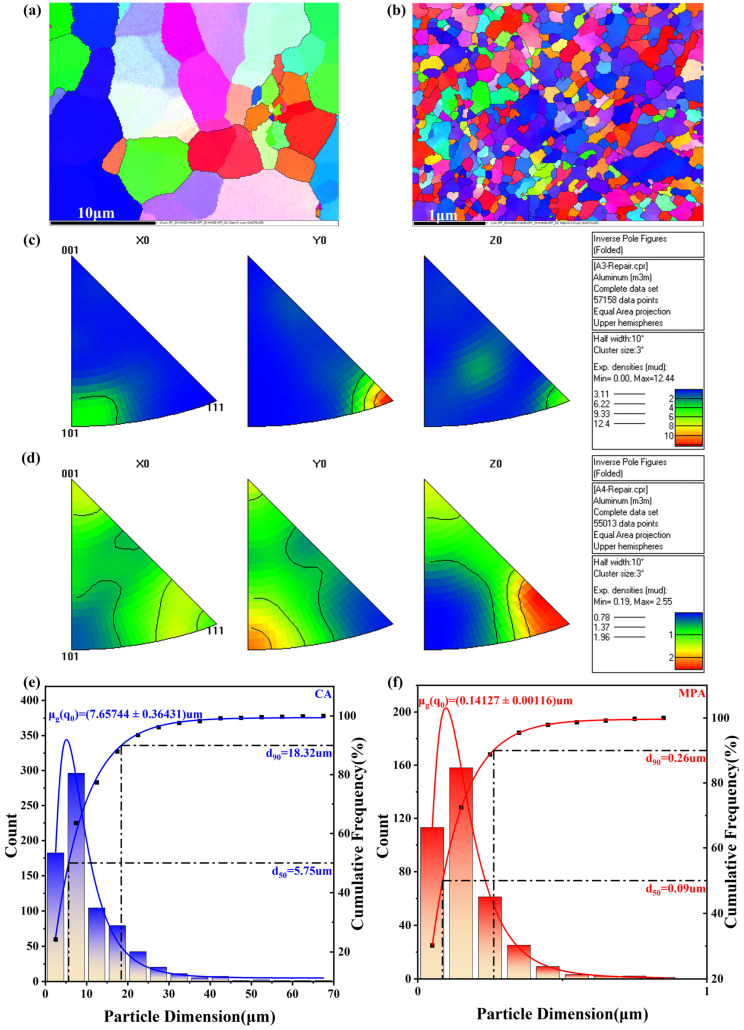
(**a**) IPF-Z0 plots of CA, (**b**) IPF-Z0 plots of MPA, (**c**) antipodal plots of CA, (**d**) antipodal plots of MPA, (**e**) grain-size statistics of CA, (**f**) grain-size statistics of MPA.

The grain size and orientation of CA and MPA were observed separately using TKD. Upon comparing Figure 5a,b, it is evident that the grain size of CA varies significantly, with most grains larger than 10 μm. In contrast, MPA exhibits minimal variation in grain size, with most grains measuring less than 300 nm. After measuring the grain sizes of both materials, the results are presented in Figure 5e,f. The D_90_ of CA grains is 18.32 μm, while the D_90_ of MPA grains is 260 nm. This substantial difference highlights the pronounced grain-refining effect. Figure 5c,d display the antipodal plots of CA and MPA, respectively. It is shown that there are noticeable differences in grain orientation and orientation density between the two materials. The grains of CA exhibit a <111> orientation parallel to the *y*-axis, with a higher orientation density. This can lead to significant strength differences in various directions of the alloy. In contrast, the grains of MPA show a <111> orientation parallel to the *z*-axis, with a lower orientation density, resulting in more balanced performance across different directions and less pronounced strength variation in the alloy.

**Figure 6:**
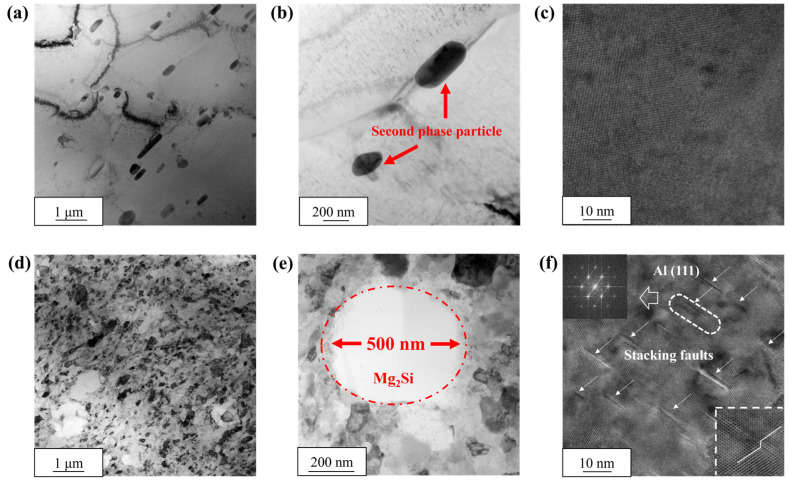
(**a**,**b**) TEM bright-field images of CA, (**d**,**e**) TEM bright-field images of MPA, (**c**) HRTEM images of CA, (**f**) HRTEM images of MPA.

CA and MPA were observed and compared separately using TEM. Figure 6a,b illustrate that the grain size of the Al matrix in CA is large, accompanied by numerous precipitated phases ranging from 200 nm to 1 μm. Upon observation, most precipitated phases in CA appear spherical or as short rods, clearly demarcated from the Al matrix. These distinct boundaries indicate poor compatibility with the Al matrix, leading to minimal reinforcement for the aluminum alloy castings.

Figure 6d reveals that the grain distribution within the Al matrix of MPA is uniform, with an average grain size of approximately 300 nm. This finding is consistent with TKD characterization, which indicates a significantly smaller grain size compared to the Al matrix of CA, highlighting a notable grain refinement effect. It is worth mentioning that the number of grain boundaries increases with grain refinement, providing more obstacles for dislocations. The regular polygonal precipitation phases are clearly visible in Figure 6e, averaging approximately 1 μm in size. A meticulous observation and comparison show that the boundary between the precipitation phases and the Al matrix is less distinct than in CA. This suggests a better lattice match with the Al matrix, enhancing the alloy’s strength by more effectively hindering dislocation movement. The HRTEM image of MPA is presented in Figure 6f, revealing the presence of high-density SFs within the Al matrix. These SFs serve a dual purposes: they increase the defect energy of the alloy, improving its overall deformation resistance; they also act as barriers to dislocation movement, further strengthening the alloy.

In summary, compared to CA, MPA exhibits a smaller grain size in the Al matrix, better lattice matching between the precipitated phase and Al matrix, and the presence of high-density SFs in the Al matrix. Importantly, in this study, both CA and MPA were prepared without artificially adding other alloying elements or rare earth elements that are not part of the Al-Mg-Si alloy.

**Figure 7:**
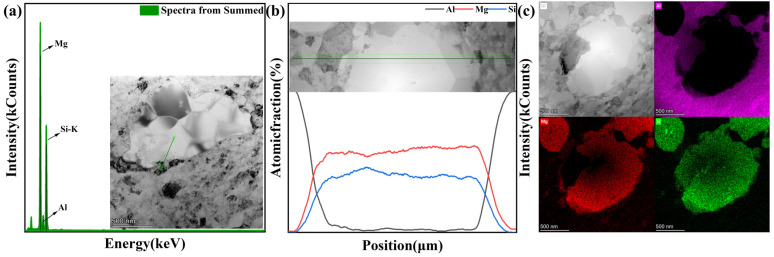
(**a**) The compositional data of EDS-spot scanning (the inset shows the spot scanning area), (**b**) the elemental line distribution results of EDS-line scanning (the inset shows the line sweep area), (**c**) the area of EDS mapping scanning and the elemental surface distribution results of the area.

Through the EDS detection of the precipitated phase in the MPA specimen, the results are shown in Figure 7a–c. The precipitated phase consists solely of two elements, Mg and Si. According to Figure 7a,b, it is evident that the ratio of Mg to Si is approximately 2:1, which coincides with the ratio of Mg to Si in Mg_2_Si. Combined with Figure 7c, it is clear that the precipitated phase is attributed to the Mg_2_Si precipitation-reinforced phase, which is characteristic of Al-Mg-Si alloys [32].

### 3.3. Mechanical Properties

**Figure 8:**
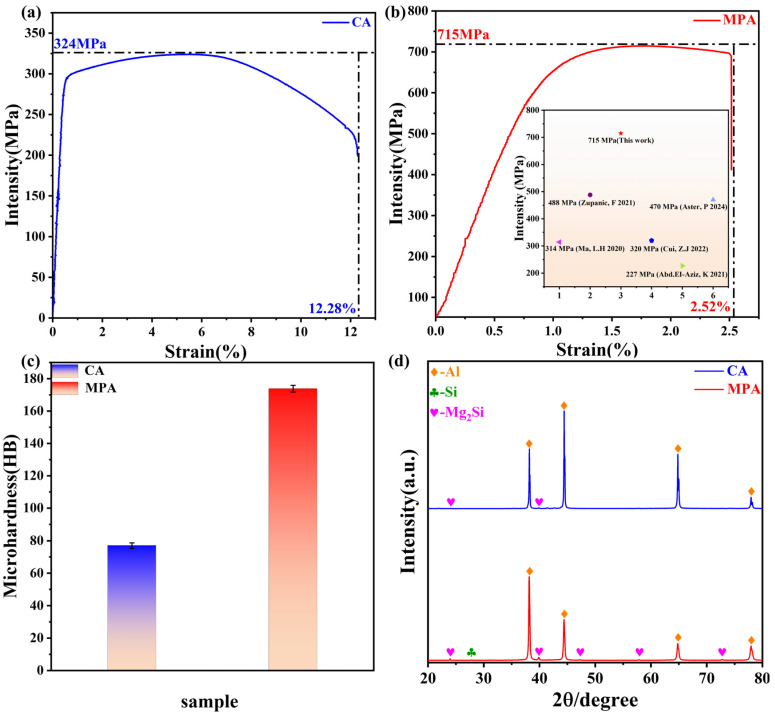
(**a**) Tensile strength and elongation of CA, (**b**) tensile strength and elongation of MPA (as well as the tensile strength of Al-Mg-Si alloys in other studies) [33,34,35,36,37], (**c**) comparison of hardness test of CA and MPA, (**d**) typical XRD spectra of CA and MPA.

Figure 8a,b depict the tensile strength and elongation of CA and MPA, respectively. As shown in Figure 8a, CA exhibits a tensile strength of 324 MPa and an elongation of 12.28%. In contrast, Figure 8b demonstrates that MPA achieves a tensile strength of 715 MPa with an elongation of 2.52%. This signifies a 2.21-fold increase in tensile strength compared to CA, albeit with a decrease in elongation. The tensile strength of this work is also much higher than that found in other studies, as shown in the illustration in Figure 8b [33,34,35,36,37]. Furthermore, the hardness of CA and MPA was evaluated and is presented in Figure 8c. The hardness of CA is 77 HB, while MPA exhibits an average hardness of 173 HB, which is approximately 2.25 times greater than that of CA. Both the hardness and tensile strength of MPA are significantly enhanced compared to CA, although there is a slight reduction in plasticity.

Figure 8d displays the XRD patterns of CA and MPA, revealing common diffraction peaks corresponding to Al and Mg_2_Si. The primary distinction lies in the notably higher content of Mg_2_Si in MPA compared to CA. Additionally, the presence of diffraction peaks corresponding to Si in MPA indicates that partially free Si remains as secondary-phase reinforcing particles in MPA. Upon closer inspection, it becomes apparent that the half-peak width of the Al peak in MPA is broader than that in CA, as described by Scheller’s formula [38]. This finding further substantiates the conclusion that the grain size of MPA is finer than that of CA.

**Figure 9:**
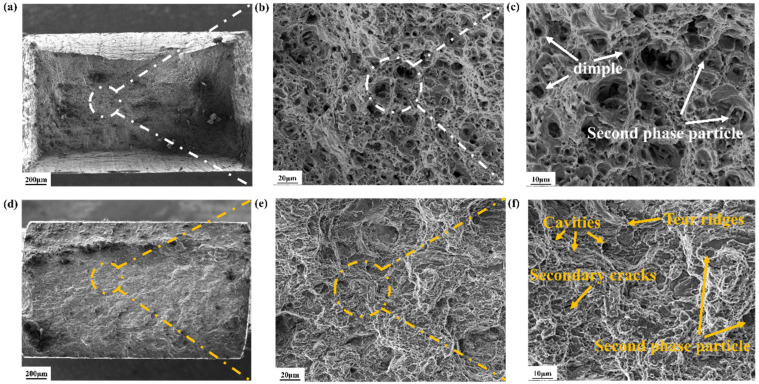
Fractographs of tensile specimen, (**a**–**c**) tensile fracture morphology of CA, (**d**–**f**) tensile fracture morphology of MPA.

To further understand the failure mechanism of the two alloys, the tensile fracture surfaces of both the CA specimen and the MPA specimen were characterized using SEM. Figure 9 presents a comparison of the fractographs of the tensile test specimens of the CA and the MPA specimens. For the fracture morphology at different magnifications in Figure 9a–c, we observe second-phase reinforcing particles in the CA specimen, which constitute the primary mode of reinforcement. Additionally, a significant number of tough dimples are clearly visible, a typical feature of ductile fracture [39]. Figure 9d–f depict the tensile fracture morphology of the MPA specimen. The images reveal a substantial presence of second-phase particles. Moreover, secondary cracks are apparent within the Si particles, attributed to stress concentration during plastic deformation. As stretching progresses, Si particles undergo cleavage fracture, resulting in numerous smooth and flat cleavage surfaces. The fracture surface also displays numerous tear ridges within the second phase and at the junctions of Si particles with the Al matrix. Upon closer inspection, voids are found on the fracture surface, caused by the debonding of second-phase particles. Overall, the MPA specimen exhibits brittle fracture at the macroscopic level and ductile fracture at the microscopic level.

## 4. Conclusions

In this study, a high-strength Al-Mg-Si alloy was fabricated using the MA and PF processes without any adjustment to alloy components. The microstructures and mechanical properties were investigated and compared to those of cast Al-Mg-Si alloys. The following conclusions can be drawn:

(1) The tensile strength of the CA sample was 324 MPa at room temperature, whereas the MPA sample exhibited superior performance with a tensile strength of 715 MPa.
(2) XRD characterization confirmed the presence of the Mg_2_Si precipitate phase in both the CA and MPA samples. However, the MPA sample exhibited a higher Mg_2_Si content compared to the casting alloys. Additionally, Si monomers were identified as second-phase reinforcing particles in the MPA sample.
(3) TKD analysis indicated that the grain size of the Al matrix in the CA sample was approximately 18.32 μm, whereas in the MPA sample, it was about 260 nm, signifying a substantial grain refinement compared to the CA sample.
(4) TEM analysis revealed the absence of high-density SFs in the CA sample, whereas significant high-density SFs were observed in the MPA sample.

The strengthening mechanisms observed in MPA primarily include grain refinement, Mg_2_Si precipitation reinforcement, and stacking fault strengthening.

## Data Availability

The original contributions presented in this study are included in the article. Further inquiries can be directed to the corresponding authors.
